# 

**Published:** 1906-12

**Authors:** 


### BOOKS RECEIVED.

Practical Dermatology. A condensed manual of diseases of the skin. By Bernard Wolff, M.D., Clinical Professor of Diseases of the Skin in the Atlanta College of Physicians and Surgeons. Large 8 vo.. pp. 288. Illustrated. Chicago: Cleveland Press. 1906.

---

Diseases of the Stomach. By Max Einhorn, M.D., Professor of Clinical Medicine at the New York Post-Graduate Medical School and Hospital. Fourth revised edition. Octavo, pp. 559. Illustrated. New York: William Wood & Co. 1906. (Price, $3.50).

---

Modern Clinical Medicine. Vol. III. Diseases of the Digestive System. Edited by Frank Billings, M.D., Professor of Medicine, University of Chicago; Professor of Medicine and Dean of Faculty, Rush Medical College. Octavo, pp. xvi-824. With 45 illustrations. New York and London: D. Appleton & Co. 1906.

---

Surgery. Its Principles and Practice. By various authors. Edited by William Williams Keen, M.D., Professor of the Principles of Surgery and of Clinical Surgery in Jefferson Medical College, Philadelphia. In 5 volumes. Volume I. History, Surgical Physiology, Surgical Pathology, Infections, Tumors, Wounds. Large Octavo, pp. 983. With 261 text illustrations and 17 colored plates. Philadelphia and London: W. B. Saunders Co. 1906. (Price, cloth, $7.00; half morocco, $8.00, net prices).

---

Blakiston's Quiz Compends. Genitourinary Diseases and Syphilis. By Charles S. Hirsch, M.D., Assistant in the Genitourinary surgical department, Jefferson Medical College. 12mo., pp. 351. Illustrated. Philadelphia: P. Blakiston's Son & Co. 1906. (Price, $1.00).

---

A Syllabus of Materia Medica. Compiled by Warren Coleman, M.D., Professor of Clinical Medicine in Cornell University Medical College. Third edition. pp. 186. New York: William Wood & Co. 1906. (Price, $1.00).

A Treatise on the Motor Apparatus of the Eyes. Embracing an Exposition of the Anomalies of the Ocular Adjustments and their Treatment, with the Anatomy and Physiology of the Muscles and their Accessories. By George T. Stevens, M.D., Ph.D. Illustrated with 184 engravings, some in colors. 496 pages, Royal Octavo. Philadelphia: F. A. Davis Company. 1906. (Price, $4.50 net).

---

A Primer of Psychology and Mental Disease for use in Training Schools for Attendants and Nurses and in Medical Classes, and as a ready reference for the practitioner. By C. B. Burr, M.D., Medical Director of Oak Grove Hospital (Flint, Mich.) for Mental and Nervous Diseases. Third edition. Revised, with illustrations. Pages viii-183, 12mo. Philadelphia: F. A. Davis Company, (Price, $1.25 net).

---

The Ear and its Diseases. A textbook for students and physicians. By Seth Scott Bishop, B.S., M.D., LL.D., Honorary President of the Faculty and Professor in the Post-Graduate School and Hospital of Chicago; Surgeon to the Post-Graduate Hospital and to the Illinois Hospital, etc. Illustrated with 27 Colored Lithographs and 200 Additional Illustrations. Royal Octavo, 440 Pages. Philadelphia: F. A. Davis Company. (Price, $4.00 net).

---

Saunders' Pocket Medical Formulary. By William M. Powell, M.D., author of "Essentials of Diseases of Children." Containing 1831 formulas from the best known authorities. With an appendix containing Posologic Tables, Formulas and Doses for Hypodermic Medication, Poisons and their Antidotes, Diameters of the Female Pelvis and Fetal Head, Obstetric Table, etc., etc. Eighth Edition, Adapted to the new (1905) Pharmacopeia. Philadelphia and London: W. B. Saunders Company, 1906. In flexible morocco, with side index, wallet and flap. ($1.75 net).

---

Prevalent Diseases of the Eye. By Samuel Theobald, M.D., Clinical Professor of Ophthalmology and Otology, Johns Hopkins University. Octavo of 551 pages, with 219 text-illustrations, and 10 colored plates. Philadelphia and London: W. B. Saunders Company. 1906. (Cloth, $4.50 net; half morocco $5.50 net).

---

A Textbook on the Practice of Gynecology. By W. Easterly Ashton, M.D., LL.D., Professor of Gynecology in the Medico-Chirurgical College of Philadelphia. Third Edition, thoroughly revised. Octavo of 1096 pages, with 1057 original line drawings. Philadelphia and London: W. B. Saunders Company, 1906. (Cloth, $6.50 net; half morocco, $7.50 net).

---

Abdominal Operations. By B. G. A. Moynihan, M.S. (London), F.R.C.S., Senior Assistant Surgeon at Leeds General Infirmary, England. Second Revised Edition, greatly Enlarged. Octavo of 815 pages, with 305 original illustrations. Philadelphia and London: W. B. Saunders Company, 1906. (Cloth, $7.00 net; half morocco, $8.00 net).

---

The American Illustrated Dictionary. All the terms used in Medicine, Surgery, Dentistry, Pharmacy, Chemistry and kindred branches; with over 100 new tables. By W. A. Newman Dorland, M.D., Fourth Revised Edition. Octavo of 836 pages, with 293 illustrations, 119 of them in colors. Philadelphia and London: W. B. Saunders Company, 1906. (Flexible Morocco, $4.50 net; thumb indexed, $5.00 net).

Diet in Health and Disease. By Julius Friedenwald, M.D., Clinical Professor of Diseases of the Stomach in the College of Physicians and Surgeons, Baltimore; and John Ruhrah, M.D., Clinical Professor of Diseases of Children in the College of Physicians and Surgeons, Baltimore. Second Revised Edition. Octavo of 728 pages. Philadelphia and London: W. B. Saunders Company, 1906. (Cloth, $4.00 net; half morocco; $5.00 net).

---

Photoscopy (Skiascopy or Retinoscopy). By Mark D. Stevenson, M.D., Ophthalmic Surgeon to the Akron City Hospital; Oculist to the Children's Home, Akron, Ohio. Octavo of 126 pages, illustrated. Philadelphia and London: W. B. Saunders Company, 1906. (Cloth, $1.25 net).

---

Obstetrics for Nurses. By Joseph B. DeLee, M.D., Professor of Obstetrics in the Northwestern University Medical School, Chicago. Second Revised Edition. 12mo., 510 pages, illustrated. Philadelphia and London: W. B. Saunders Company, 1906. (Cloth, $2.50 net).

---

The Technic of Operations upon the Intestines and Stomach. By Alfred H. Gould, M.D., Boston, Massachusetts. Octavo volume, containing 190 beautiful original illustrations, some in colors. Philadelphia and London: W. B. Saunders Company, 1906. (Cloth, $5.00 net; half morocco, $6.00 net).

---

A Textbook of Obstetrics. By Barton Cooke Hirst, M.D., Professor of Obstetrics in the University of Pennsylvania. Fifth Revised Edition. Octavo of 915 pages, with 753 illustrations, 39 in colors. Philadelphia and London: W. B. Saunders Company, 1906. (Cloth, $5.00 net; half morocco, $6.00 net).

---

Studies in the Psychology of Sex—Erotic Symbolism, The Mechanism of Detumescence, The Psychic State of Pregnancy. By Havelock Ellis. Pages x-285. Sold only by Subscription to Physicians, Lawyers, and Scientists. F. A. Davis Company, Philadelphia. ($2.00 net).

---

Transactions of the American Otological Society. Thirty-ninth annual meeting held at New York, June 26 and 27, 1906. F. E. Jack, M.D., Boston, Secretary.

---

The Practical Medicine Series of Year Books. Ten volumes. Series of 1906. Issued under the general editorial charge of Gustavus P. Head, M.D., Professor of Laryngology and Rhinology in the Chicago Post-Graduate Medical School. Vol. VII. Pediatrics and Orthopedic Surgery, edited by Drs. Isaac A. Abt and John Ridlon. Chicago: The Year Book Publishers. Series, 1906. (Price, $1.25; entire series, $10.00).

---

Retinoscopy in the Determination of Refraction at One Meter distance with the Plane Mirror. By James Thorington, M.D., Author of "Rrefraction and How to Refract," etc. Fifth edition. pp. 67. With 54 illustrations. Philadelphia: P. Blakiston's Son & Co. 1906.

---

The Medical Directory of New York, New Jersey and Connecticut. Published by the Medical Society of the State of New York. Volume VIII. 1906.

---

Genitourinary Diseases and Syphilis. By Henry H. Morton, M.D., Clinical Professor of Genitourinary Diseases in the Long Island College Hospital. Illustrated with 158 Half-tone and Photoengravings and 7 Full-page colored plates. Second Edition, Revised and Enlarged. Royal Octavo, 500 Pages. Philadelphia: F. A. Davis Company. 1906. (Price, $4.00 net).

---

Atlas and Textbook of Human Anatomy. Volume I. By Professor J. Sobotta, of Wurzburg. Edited, with additions, by J. Playfair McMurrich, A.M., Ph.D., Professor of Anatomy at the University of Michigan, Ann Arbor. Quarto volume of 258 pages, containing 320 illustrations, mostly in colors. Philadelphia and London: W. B. Saunders, 1906. (Cloth $6.00 net; half morocco, $7.00 net).

---

Medical Epitome Series. Edited by Victor C. Pederson, M.D., Lecturer in surgery in the New York Polyclinic Medical School and Hospital. Pathology, by John Stenhouse, M.D., and John Ferguson, M.D. 12 mo. pp. 285. Illustrated. Philadelphia and New York: Lea Bros. & Co. (Price, $1.00).

